# A structural model of the profilin–formin pacemaker system for actin filament elongation

**DOI:** 10.1038/s41598-022-25011-w

**Published:** 2022-11-28

**Authors:** Clarence E. Schutt, Mattias Karlén, Roger Karlsson

**Affiliations:** 1 Department of Chemistry, Princeton University, Princeton, NJ USA; 2 Firma Mattias Karlén, Stockholm, Sweden; 3 Department of Molecular Biosciences, WGI, Stockholm University, Stockholm, Sweden

**Keywords:** Structural biology, Cell biology, Cytoskeleton, Actin

## Abstract

The formins constitute a large class of multi-domain polymerases that catalyze the localization and growth of unbranched actin filaments in cells from yeast to mammals. The conserved FH2 domains form dimers that bind actin at the barbed end of growing filaments and remain attached as new subunits are added. Profilin–actin is recruited and delivered to the barbed end by formin FH1 domains via the binding of profilin to interspersed tracts of poly-l-proline. We present a structural model showing that profilin–actin can bind the FH2 dimer at the barbed end stabilizing a state where profilin prevents its associated actin subunit from directly joining the barbed end. It is only with the dissociation of profilin from the polymerase that an actin subunit rotates and docks into its helical position, consistent with observations that under physiological conditions optimal elongation rates depend on the dissociation rate of profilin, independently of cellular concentrations of actin subunits.

## Introduction

In eukaryotes, vital processes such as cell growth, migration and surface motility all require polymerization of actin, a highly conserved protein ATPase. This process is regulated by actin polymerases, known as formins, that respond to second messenger GTPases by churning out filaments that can be free, branched or bundled into myriad tension-bearing structures by a host of actin binding proteins^1,2^. An outstanding issue for structural cell biology is to explain how multiple classes of formins serving in these widely diverse roles optimize their use of a common pool of actin monomers in living cells.

The multi-domain formins have processive ‘leaky caps’^3,4^ remaining bound as actin subunits are added at the barbed end of filaments in a wide variety of essential biological processes, including filopodial extension, establishment of polarity, and cytokinesis, reviewed in^5^. Pools of cytoplasmic actin are maintained by the highly-abundant monomer-sequestering protein profilin^6^ which binds poly-l-proline on an aromatic surface patch that is exposed even when profilin is bound to actin^7–10^, endowing 1:1 complexes of profilin–actin with the ability to bind short tracts of (~ 10 residues) poly-l-proline in formin FH1 domains, acting to concentrate and feed profilin–actin to the formin FH2 domains, which carry out the core function of adding actin monomers at the barbed end^11–16^.

A much studied, but unresolved, problem is to explain in molecular terms how the transfer of actin monomers from profilin to the barbed end of filaments is governed by the FH2 domains of formin polymerases. Crystal structures of dimers of the actin-binding FH2 domain from budding yeast formin (Bnil) have revealed how the highly flexible ‘linker-lasso’ at the N-terminal end of the domain binds the C-terminal ‘post’ of the coiled-coil actin-binding bridge element of its dimer-related binding partner^17^ to form a ring. Although the lasso does not bond with the post of its symmetry-related FH2 subdomain in Bni1 FH2–actin co-crystals, it does bind to the post of another FH2 molecule related by translational symmetry, generating a helical ramp around a central core of actin molecules bound to the knobs of the FH2 domains. The flexibility of the linker^18^ suggested the possibility that crystal packing may have forced a ‘domain-swap’^19^, breaking the supposed ‘natural’ ring structure as seen in the crystal structure of the Bni1 FH2 actin-free dimer^18^. However, even though the two bound actin molecules are related by a 27.5 Å rise per subunit (as in actin filaments) in Bni1 FH2–actin co-crystals they are too far apart and untwisted by 13° to be part of an actual actin filament. Nevertheless, this crystal packing model has given rise to the class of ‘stair-stepping’ models for filament elongation where each FH2 molecule in turn steps forward to bind a successive actin molecule^18,20^.

The crystal structure of the mammalian formin FMNL3 FH2–actin complex^21^ poses a different question for structural analysis because the two bound actin molecules are related by a quasi-two-fold axis that bears no obvious relationship to the actin helix. However, it was observed that sliding one molecule past the other by 20 Å would bring both actin molecules into plausible positions on opposite strands about a putative filament axis, allowing binding without clashes for an incoming actin molecule at the barbed end. The FH2 actin-binding knob and post regions in the Bni1 FH2–actin and FMNL3 FH2–actin structures individually superpose well, but the knob domains bind actin at different angles in the two structures resulting in a relative rotation of the coiled coil and distal post domains^21^.

The availability of atomic resolution cryo-electron microscope structures of actin filaments^22,23^ invites reconsideration of molecular models for the formin elongation reaction, in particular the precise role of profilin in delivering actin subunits to the barbed end. Based on an analysis of the Bni1–actin and FMNL3–actin structures with respect to docked actin filaments we present a variation of the staircase stepping model wherein profilin not only delivers actin to the barbed end but remains bound to the polymerase, playing a critical role in the timing of the reaction. The presence of profilin bound to the terminal actin subunit breaks the quasi-two-fold symmetry relating the two actin molecules, creating a barrier to the rapid interconversion between closed (2-fold screw symmetric) and open (helically symmetric) states that is a feature of currently accepted structure-based staircase stepping models^24^. The release of profilin from the polymerase results in a 13° rotation and docking of its associated actin molecule into its helical niche along with dissociation and translocation of the FH2 molecule on the opposite strand to a position where it can accept a new incoming profilin–actin complex. This model is thus an elaboration of the ‘stepping second’ model proposed to explain elegant experiments on single filaments growing in vitro under the real-time control of engineered FH1-FH2 constructs^16^.

Recently, it has been demonstrated under in vivo conditions, where concentrations of profilin–actin are up to ten times higher, that the rate of the elongation reaction follows simple Michaelis–Menten kinetics in the concentration of profilin^25^. It is surprising, given all the rate constants involved, that the rate of profilin release from the polymerase alone dictates optimal performance of formins at saturating concentrations of actin^25^. This ‘pacemaker’ role for profilin can be understood in terms of the structure-based model presented here.

## Results

In the Bni1–actin co-crystal structure, the FH2 domains spiral around a core of actin molecules related by a 2-fold screw axis of symmetry^17^. The FH2–actin heterodimers are equivalent (in identical crystalline environments). However, in a dimer derived from the crystal structure, the heterodimers are no longer equivalent because the linker-lasso of one of the FH2 molecules is not bound to the adjacent post of the other FH2 molecule. In models where the ring is manually closed, the imposed two-fold screw symmetry of the crystal-derived ring must be reconciled with the helical symmetry of the bound filament, giving rise to a search for intrinsic ‘gating factors’ that control access of the incoming actin subunit to the helical barbed end^12,24,26^.

**Figure 1:**
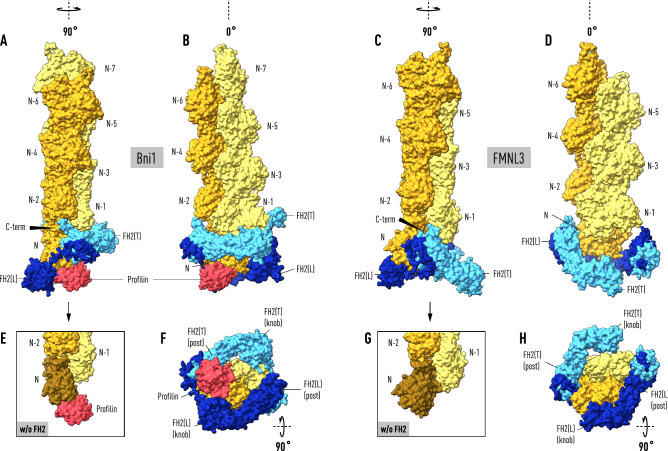
Actin filaments docked onto formin:actin heterodimers support a ‘stepping second’ model. The two panels on the left depict the result of superimposing the terminal subunit of a 6-mer (in lemon and orange) of the helical actin filament (PDB code: 6DJO) onto actin molecule (N−1) bound to the trailing FH2(T) domain (light blue) in the Bni1:actin heterodimer (PDB code: I64Y). With this alignment, actin(N) bound to FH2(L) (in dark blue) does not fit the helical symmetry of the filament but is instead coplanar with actin(N−2). Actin subunits in the filament are not N-acetylated. Superposing β-actin from the crystal structure of profilin-β-actin (PDB code: 2BTF) onto actin(N) places profilin (in red) in a shallow surface pocket where it forms significant salt-bridges with FH2(L) and the N-terminus of actin(N). The coplanarity of actin(N) (in brown) with actin(N−2) is seen in the bottom panels (**E**,**G**) where FH2 has been removed. The two panels on the upper right show the terminal subunit of a 6-mer of the helical actin filament docked onto actin(N) of the mammalian formin FMNL3:actin crystal structure (PDP code: 4EAH). For consistency in relating the Bni1 and FMNL3 filament-bound structures to a second stepping model, the FH2 domains are labeled so that actin(N) is bound to FH2(L) on the right even though it is not the ‘leading’ FH2 domain. Not shown in the panels on the right is the actin molecule bound to FH2(T) in the crystal structure which is in a ‘jammed’ position evidently caused by front-to-back packing between the two ‘biological units’ in the asymmetric unit (see “Methods”). Alignment of the filament axes in the Bni1 (left panels) and FMNL3 models (right panels), discloses a two-state ‘stepping second’ model in which FH2(T) descends towards the barbed end to create an open pocket for the binding of actin(N+1) with the dissociation of profilin from actin(N). The arrows (panels **A** and **C**) point to the prominent α-helix at the C-terminus (C-term) of FH2(T) at the interface between actin(N−2) and actin(N).

To model the Bni1–actin formin cap we applied a C2_1_ symmetry operation to the asymmetric unit of the crystal structure (PDB code: 1Y64) to form a dimer of heterodimers with one closed end, where the lasso-post interaction is preserved, and an open end where the linker-lasso is not bound to the post of its quasi-symmetry-related FH2 molecule. We found that superposing the filament structure (PDB code: 6DJO) onto the actin molecule bound to the trailing FH2(T) results in a filament axis at an angle of 13° to the crystal 2-fold screw axis relating the heterodimers (Fig. 1). Notably, actin(N) bound to FH2(L) is not in the correct orientation relative to actin(N−1) on the opposite strand to be part of the superposed helical filament. In fact, subdomains 1 and 2 (without residues 40–51) of actin(N) are coplanar with actin(N−2) on the same strand. This is demonstrated by the experimentally determined filament direction cosines (direction cosines: α = 93.1°_,_ β = 12.9°, χ = 102.3°, see “Methods”), which are close to the values (90°, 13°, 103°) for exact coplanarity given the 2-fold screw symmetry of the polymerase and the 27.5 Å rise per subunit.

Similarly, the actin molecule bound to FMNL3 FH2(L) (in our labeling scheme) has the correct orientation relative to the crystal axis of symmetry to fit the helical geometry of the filament, implying that the crystal symmetry and filament axes are collinear^21^. Superposing the actin filament structure (PDB code: 6DJO) on the actin subunit bound to FH2(L) in the FMNL3 structure discloses the putative axial position of actin(N+1). This position does not overlap with the actin molecule bound to FH2(T) but is shifted axially towards the barbed end. Aligning the Bni1 and FMNL3 models supports a two-state ‘stepping second’ model^16^ where FH2(T) migrates to the barbed end to engage actin(N+1) as actin(N) rotates and docks into its helically symmetric position in the filament (Fig. 1).

**Figure 2:**
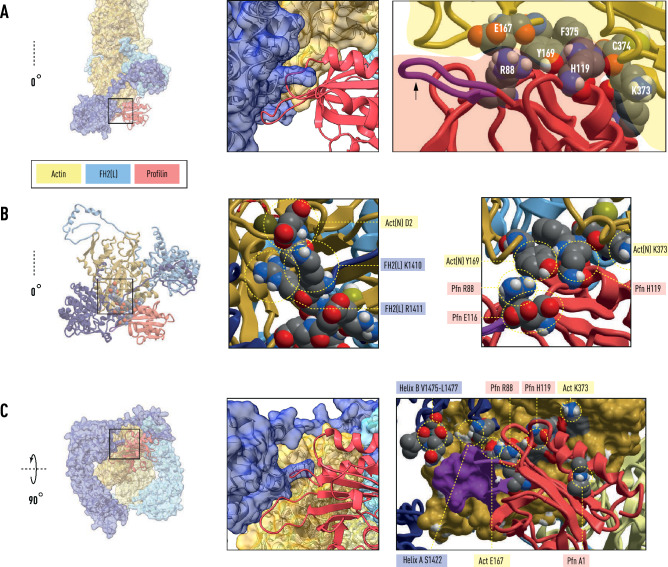
Central role for profilin R88 in coordinating actin polymerization by FH2. The boxed region of panel (**A**) shows profilin residues R88 and H119 sequestering Y169, the keystone residue in the longitudinal actin(N)-actin(N−2) interface. Actin residues K373, C374 and F375 form part of the pocket surrounding Y169. The arrow points to the prominent profilin ‘wing’ R88-T97 (purple). Panel (**B**), boxed region, shows (left) the interaction between actin(N) residue D2 and the FH2(L) linker residues K1410 and R1411. Detailed view (right image of panel **B**) of profilin–actin interface shows the salt-bridge between R88 and profilin residue E116 and an electrostatic interaction involving actin residues Y169, K372 and profilin residue H119. The boxed region of panel (**C**) shows the sidechain of profilin R88, held in position by a salt-bridge with E167 of actin(N), lying along the base of the profilin ‘wing’ (purple: R88–T97) that contacts the turns at the ends of the actin-binding FH2 knob helices (**A**) and (**B**).

**Figure 3:**
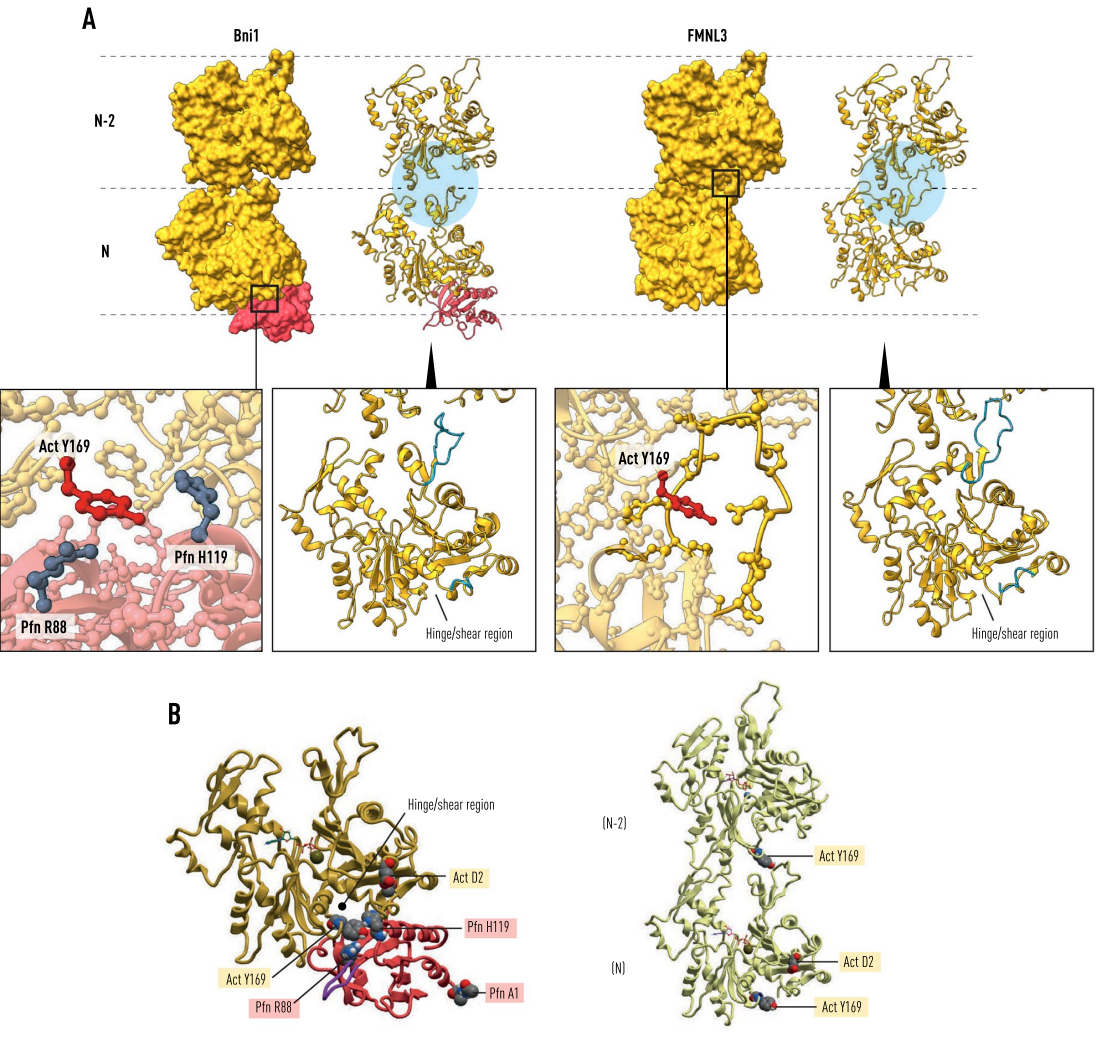
Profilin release associated with polymerization. Panel (**A**) shows actin(N)-actin(N−2) as bound by the Bni1 and FMNL3 FH2 dimers, (left) and (right), respectively. In Bni1, the actin molecules are coplanar, while in FMNL3 they are related by the helical symmetry of the cryo-EM structure (PDB: 6DJO). During the release of profilin from actin(N) in the Bni1 structure the D-loop in SD2 extends towards actin(N−2) as seen in the FMNL3 structure. Y169, the central residue in the longitudinal contact between actin(N) and actin(N−2), is exposed with the release of profilin. Panel (**B**) depicts the profilin-β-actin structure (PDB: 2BTF) showing the proximity of the profilin binding site to the hinge and shear region of actin. The FH2 knob helix A occupies part of this site with profilin (not shown). The right image illustrates the environment of Y169 in the longitudinal bond of the filament.

We propose that profilin–actin(N+1) is held in a vise-like grip by the knob domain of the descended FH2(T) and the post domain of FH2(L). The binding of the lasso to the post of FH2(L) seals the closed end, resulting in the formation of the electrostatic bond between the N-terminus of actin(N+1) and the FH2(T) linker. The prominent profilin ‘wing’ (R88–T97) interacts with the N-terminal end of knob helix A (residues 1422–1440) and the C-terminal end of knob helix B (residues 1457–1479), with profilin residue R88 playing a central coordinating role (Fig. 2). The key structural determinants of polymerization (Y169, D2–D4, C374–F375 of actin) are protected by the binding of profilin to actin(N) (Figs. 2 and 3). As these events ensue, the same set of interactions is broken at the opposite end of the polymerase with the dissociation of profilin resulting in actin(N) annealing into the helical filament.

**Figure 4:**
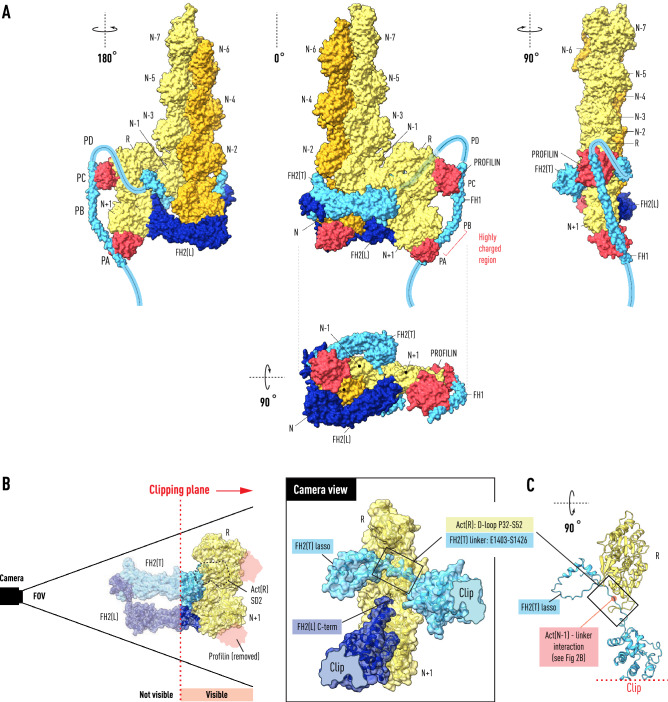
Transition State for the Formin Elongation Reaction. The Bni1:actin FH2 heterodimer with bound F-actin (left in Fig. 1) is shown with a profilin–β-actin dimer derived from the crystal structure (space group 19; PDB: 2BTF) docked onto the penultimate actin(N−1) subunit. The center panel in (**A**) illustrates how the binding of the dimer positions actin(N+1) at the entrance to the polymerase at the open end with the axial spacing (5.5 nm) of the actin helix. The PA and PC poly-l-proline tracts of FH1 have been docked onto profilins bound to actin(N+1) and actin(R) respectively. The path (faint blue, middle panel) of the FH2(T) linker (1400–1420) in the open space below the actin(N−1)–actin(R) interface is dictated by its interaction with the actin(N−1) N-terminus, positioning the lasso on the opposite side of the dimer from its knob domain. Panel (**A**) show in three orientations a possible path for the FH1 polypeptide as it emerges (in the carboxy to amino direction) from the top of the FH2(T) lasso. Panel (**B**) shows the transition-state in the same orientation as the central panel of (**A**) but clipped by a plane the cuts through the center of the structure removing from view actin(N−1) and most of FH2(T) and FH2(L), except for parts of the knob and post domains respectively. The camera view (center), along a line perpendicular to the clipping plane, shows the space occupied by the FH2(T) linker and lasso. The negatively charged C-terminal α-helix of the post of FH2(L) is shown in the vicinity of the positively charged linker. Panel (**C**) shows selected residues of the clipped structure from the top to reveal the position (red dot) of a three-way interaction between the linker, the N-terminus of actin(N−1) and the D-loop of actin(R).

In terms of the kinetic scheme inferred for polymerase activity at the high concentration of profilin–actin^25^ found intracellularly this corresponds to the very fast step in the elongation reaction. In our model, this results from the formation of the extensive longitudinal bond between actin(N) and actin(N−2) as the terminal actin subunit enters the helix (Fig. 3). By analogy to a transition-state in an enzymatic reaction, we devised a mechanism for guiding the incoming profilin–actin into the coplanar position (Fig. 4). Dimers are the predominant oligomeric species in solutions of polymerizing actin molecules^27^ and non-polymerizable dimers (subunits not related by helical symmetry) are present in weight fractions equal to those of helical dimers^28^, which led us to consider how the binding of a dimer of profilin–actin might facilitate the establishment of the coplanar barbed end. In the proposed transition-state, three actin molecules are held together in a right-angled flat structure stabilized by the pattern of oligomeric-like bonds found in profilin–actin crystals^9^ (Fig. 4). The bridging subunit actin(R) constrains the incoming profilin–actin(N+1) to be in the same plane as the penultimate actin(N−1), positioning it to slide into its binding site between the knob of FH2(T) and the post of FH2(L).

It is well-established that profilin accelerates formin-mediated polymerization^29,30^ by delivering FH1-bound profilin–actin to the barbed end^11–14^. To investigate the role of FH1 in delivering profilin–actin dimers to the barbed end we first carried out a series of homologous substitutions on the profilin–actin molecules in the transition-state structure to load the profilins with poly-l-proline peptides. The Bni1-FH1 sequence contains two long poly-l-protein tracts (PA and PB with 11 and 13 prolines, respectively) and two shorter tracts (PC and PD with 7 and 4 proline residues, respectively) (Fig. 4). Based on molecular dynamics studies^31,32^ and in the absence of a determined structure or homology model, we initially chose a straight helical rod for the 90 amino acid FH1 sequence (residues 1225–1315 containing PA, PB, PC) because of the long persistence length of poly-l-proline and the presence of a highly-charged sequence between PA and PB (–E–K–E–K–K–S–E–D–D–T–V–K–Q–E–T–T–G–D–) reminiscent of the single alpha helix domain (SAH domain) found in high abundance in the human genome^33^. Remarkably, after only a small backbone rotation in T1268 (Δφ = − 15°) at a (T–T–G) hinge between the putative SAH domain and PB, the poly-l-proline tracts PA and PC could be overlapped with the proline-rich peptides bound to profilin(N + 1) and profilin(R) respectively.

Measurements on FH1–FH2 constructs having only one PLP tract show that single tracts can support elongation rates in vitro comparable to wild-type Bni1 FH1–FH2^11^. The trend in single tract rates, higher proximal (PD) to lower distal (PA), supports a model where the collision frequency of the PLP domains on the flexible FH1 chain is determined by the distance from the barbed end. The disabled PLP domains (PB, PC, PD in the case of the PA single tract construct) in these experiments were substituted with repeated stretches of (–G–G–G–S–) that would be expected to increase FH1 flexibility, biassing the results towards the diffusion-collision model, and making it difficult to compare the results with the expectations of our ‘stiff-rod’ model with its rigid SAH-PB helix. However, the fact that PA is closest to the barbed end in our model (Fig. 4), rather than being most distal could account for ‘position-specific effects’ in double-tract experiments as discussed at page 4521 in ref.^11^.

The gateway to the knob domain at the end of the linker region is a cluster of charged amino acids (K1410, R1411, K1412, E1413, D1414) that interact with the negatively charged acetylated-N-terminus of actin. A molecular dynamics simulation^24^ of this region in the absence of profilin revealed that it can act as a sensitive electrostatic switch between the contracted and expanded states of the Bni1 FH2 dimer^17,18^. Our modeling suggests that, when profilin–actin binds to FH2, R88 stabilizes a pre-elongation state maintaining the interaction of FH2 knob helix A with the shearing interface between subdomains 1 and 3 of actin^34,35^ (Figs. 2 and 3). The profilin surface loops also cement actin residue Y169, the keystone of the longitudinal contact in actin filaments^22,23^, as well as C374 and F375, both sensitive triggers for polymerization^36,37^ associated with structural changes in subdomain 2 of actin.

Our model of the polymerase with docked F-actin reveals an intriguing ‘switching’ role for the WH2-like α-helix at the C-terminus of FH2(T)^21,38^. In the context of the ‘stepping second’ model, this structural motif, known to share a common binding site on actin with profilin, moves into position to enforce coplanarity between actin(N−2) and actin(N) until Y169 and the D-loop are engaged (see Fig. 1A,C). Furthermore, given the path of the linker in our model (Fig. 4), it appears that electrostatic interactions with the highly-charged α-helical turn at the terminus of FH2(L) mediate the binding of the FH2(T) lasso to the FH2(L) post.

**Figure 5:**
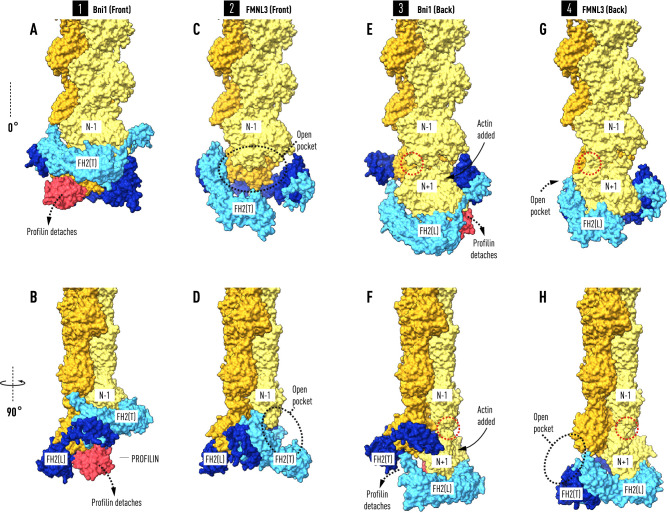
Profilin controls the timing of the elongation reaction. The front and back views of the two co-crystal structures afford views of the interaction between FH2 domains and actin at four steps (A–B, C–D, E–F, G–H) during the elongation reaction. Panels (**A**) and (**B**) show two orientations of the Bni1 FH2-actin heterodimer (modeled as in Fig. 1) stabilized by the binding of profilin–actin(N) to FH2(L). Actin(N) and actin(N−2) are coplanar. In panels (**C**) and (**D**) profilin has been released from actin(N) during the migration of FH2(T) towards the barbed end creating a binding pocket for actin(N+1). Actin(N) rotates 13° and docks into the helical niche formed by actin(N−1) and actin(N−2). In panels (**E**) and (**F**) the insertion of profilin–actin(N+1) results in the coplanar alignment of actin(N+1) with actin(N−1). In panels (**G**) and (**H**), following the release of profilin, actin(N+1) rotates and docks onto the barbed end of the filament with the closing of a small gap (indicated by red dotted circle) between actin(N−1) and actin(N+1). Note that to model the four steps in the reaction sequence as viewed from the front, the colors of FH2(L) and FH2(T), as well as those of the filament strands, have been reversed in (**E**,**F**) and (**G**,**H**) from the front view structures on the left (**A**,**B** and **C**,**D**). To model the addition of the new subunit N+1, N (not labelled) in panels (**A**,**B**) and (**C**,**D**) is relabeled N+1 on panels (**E**,**F**) and (**G**,**H**).

The binding of profilin–actin(R) to the penultimate actin(N−1) at the barbed end initiates a chain of events having a destabilizing effect on these interactions, triggering the dissociation of profilin and exposing Y169, C374 and F375 (Figs. 2 and 3). Well-studied linked conformational changes in actin itself^36,37,39^, involving changes in the N- and C-termini, the nucleotide binding cleft at meHis73^40^, and the DNase-binding loop, mediate the annealing of the leading actin(N) subunit into its three-way helical bond with actin(N−1) and actin(N−2). The D-loop in subdomain 2 of actin(R) binds the FH2(T) linker between the lasso and the electrostatic switch formed by the N-terminus of actin(N−1) and the linker (Fig. 4C). This interaction couples lasso movement to the rotation of actin(R) as actin(N+1) binds in the pocket formed by the knob of FH2(T) and the post of FH2(L). As FH2(T) descends, these linkages result in the lasso moving to engage the post and the breakage of the actin(R)–actin(N−1) interaction (Fig. 5). Longer linkers with flexible segments of gly-ser residues introduced between the D-loop and the lasso will disrupt this tight coupling. This will have the effect of decreasing the elongation rate, and offers a resolution to the paradox that shorter linkers (optimal in this case) have faster elongation rates^41^.

The crystal structure of profilin from the budding yeast *S. pombe* does not have the prominent ‘wing’ that plays a key role in the interactions described above^42^ at the closed end, and over-expression of human profilin fails to restore function to temperature-sensitive lethal profilin mutants. Although there is no crystal structure of the FH2 of cdc12p, the *S. pombe* formin, an homology model has been constructed and analyzed by molecular dynamics^24^. It appears from this model that the greater number of salt-bridges between the knob A and B helices of cdc12p and actin, compared with Bni1, and differences in sidechain packing at *S. pombe* profilin L106, which corresponds to mammalian profilin H119, endow this region with the ability to substitute for the R88-T97 ‘wing’ in gating the elongation reaction with *S. pombe* profilin.

## Discussion

Analysis of the crystal structures of the two available FH2–actin heterodimers^17,21^ discloses a common mechanistic feature of formin-catalyzed polymerization, namely that the incoming actin molecule is constrained to be coplanar with the penultimate subunit on its target strand until it becomes incorporated at the barbed end of the filament upon release of its bound profilin. Intrinsic structural features influencing the off-rate of profilin alone are sufficient to control cyclical transitions between the two-fold screw symmetry of FH2–actin heterodimers with the helical symmetry of actin filaments. This eliminates the need for postulating a rapid equilibrium between open and closed states. Indeed, in our model, the linker is the ‘gate’, and the lasso ‘unlatched from its post’ allows access to the binding pocket for the incoming profilin–actin. As FH2(T) and its linker-lasso move towards the barbed end to engage the FH2(L) post, FH1 is pulled into position to bind another profilin–actin dimer, exemplifying the integral role that FH1 plays in the elongation reaction itself, not simply acting as a delivery vehicle concentrating actin at the barbed end^13^. The precision of this transition-state mechanism and the involvement of cytoplasmic^40,43^ actin may account for the large rate difference in vivo (~ 800 subunits/s)^25^ and in vitro (~ 80 subunits/s)^13^.

**Figure 6:**
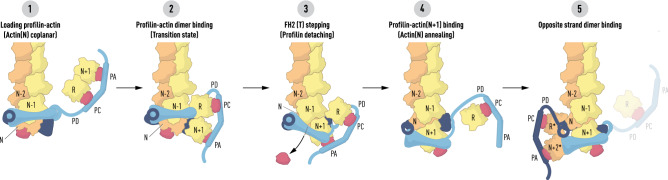
Schematic of the formin elongation reaction. Panel **1** represents the state of the actin polymerase after the insertion of actin(N) in a coplanar orientation with actin(N−2) on the same strand (orange). Its associated profilin (red) is held in place by its interaction with actin(N) and FH2(L). The actin(R)-actin(N+1) dimer has bound FH1 via the affinity of their profilin molecules for poly-l-proline tracts (PC and PA). Panel **2** depicts the binding of actin(R) to actin(N−1) which places actin(N+1) at the entrance to its binding pocket. Panel **3** shows the second stepping event where the dissociation of FH2(T) and its migration towards the barbed end ferries its linker-lasso (light blue) towards the post of FH2(L) as profilin is released from actin(N). The bond between actin(R) and actin(N−1) is broken as actin(N+1) is drawn into the pocket formed by the knob of FH2(T) and the post of FH2(L). Panel **4** represents the state where actin(N) has rotated and docked into the helical niche formed by actin(N−1) and actin(N−2). Actin(N−1) and actin(N+1) are coplanar, actin(R) has been carried off by FH1 and the FH2(T) lasso has bound to the FH2(L) post. Panel **5** illustrates step 2 in the recruitment of N+2 on the opposite strand (orange, marked *) of the actin filament after actin(R*) has bound to the (now) penultimate subunit actin(N). The FH2(T) linker-lasso and FH1 are in dark blue.

Our modelling supports a variation of the ‘stepping second’ staircase model^16^ in which each polymerase-bound actin subunit is coplanar with the previously added subunit on its target strand while remaining bound to profilin. When the profilin molecule bound to actin(N) releases in response to the binding of the profilin–actin(N+1) to the polymerase, its associated actin molecule rotates 13° as it docks into its helical niche at the barbed end of the filament, as shown in Figs. 3, 5 and 6. Previous workers have pointed out that a conformational change in actin is required for the release of profilin^16^ in stepping second models. In addition, our work suggests that the key structural event is a fast transition from a metastable coplanar state to the longitudinal helical ground state (Fig. 3).

The structural models described here were constructed by superposing well-refined and conserved crystal structures, both as individual molecules and in complex with each other and other actin-binding proteins. As such, the models can be considered as strain-free energy-minimized states. Paul and Pollard have shown that the binding of an actin subunit to the barbed end of formin-capped filaments is sufficient to drive the elongation reaction^16^. Similarly, in our model, the binding of a profilin–actin complex to the penultimate actin subunit disrupts the network of electrostatic interactions at the closed end of the polymerase and reduces the rotational entropy^44^ of the incoming actin molecule at the open end.

A novel feature of our model is that the leading actin(N) is prevented by its interaction with FH2(L) from fully bonding actin(N−1) and actin(N−2) until its bound profilin is released (Figs. 3, 5 and 6). Indeed, mutational analysis of profilin residue R88, flanking the profilin ‘wing’ (R88–T97) that bridges knob helices A and B, has led to the proposal that profilin and FH2 domains constitute a ‘pacemaker’ directly coupling the rate of actin filament elongation to profilin dissociation rates over a large range of cellular contexts and profilin–actin concentrations^25^. On the basis of this model, one might expect that microinjection of a zero-length cross-linked profilin–actin complex^43^ into motile cells would result in a complete cessation of actin-based motility^45,46^, both of which have been observed. Thus, profilin, fed continuously to the polymerase during the reaction and carried off unchanged by FH1, can be thought of as a ‘polymerization enzyme’ analogous to the description of its role during VASP-driven filament elongation, see^47^.

It is a remarkable feature of actin that it can form two kinds of polymers: the classical helical filament, and the flat ribbon-like oligomer^9,48,49^ from which a dimer can be derived that binds at right angles to the filament, forming a three-way coplanar contact. The strength of the contact stabilizes a transition state without which the incoming actin subunit would not align precisely along the reaction coordinate^50^ nor incorporate the profilin molecule essential for maintaining a constant rate of elongation under physiological conditions^25^.

The relevance of crystal packing interactions for understanding cellular function has been extensively documented and computer algorithms have been developed to systematize the interface evaluation procedure^51^. Although the actin–actin contacts in profilin–β-actin crystals meet the criteria for being valid interfaces, and were seen in the crystal structure of cross-linked trimers of actin^48^, complexed with gelsolin-subfragment-1, where helically-symmetric contacts had been expected, they have not been observed elsewhere. However, the extreme sensitivity of profilin–β-actin crystals to minute changes in the composition of the crystal bathing media, small ‘bursts’ of temperature, as well as the unusual steps required to prevent filament growth during crystal formation, led to the proposal that profilin–β-actin ribbons are metastable intermediates in an oligomeric assembly process^49^. The free energy change involved in this highly unusual and cooperative solid-state transition, clearly relevant for understanding the fast step in the elongation reaction (Figs. 2 and 3), has been measured by the direct application of osmotic force to the profilin–β-actin crystals, yielding a value of 0.05–0.1 kcal/mole of actin (~ 10% kT at 20 °C)^39^.

## Methods

The models described in this paper were arrived at by a series of superpositions involving well-resolved crystal structures of skeletal muscle actin in complex with budding yeast formin (PDB code: 1Y64) and mammalian formin (PDB code: 4EAH). All superpositions were carried out with the SPDBV program^52^, using “FIT selected residues”, followed by “FIX residues with clashes” to substitute rotamers in several sidechains (see http://www.expasy.org/spdbv/). Subsequently, molecular graphics and analyses were performed using UCSF ChimeraX^53^, and MolSoft ICM-Browser-Pro and ICM-Pro (https://www.molsoft.com/).

In the case of Bni1, we generated the FH2–actin heterodimer by applying a two-fold screw operation to the asymmetric unit (PDB code: 1Y64). We did not manually attach the lasso to the post in order to form a ring, leaving the linker in the energy-minimized state obtained by crystallography. Consequently, the FH2(T) linker follows a path along subdomain-1 of actin(N−1) that takes it to the opposite side of the polymerase from the position of its knob (Fig. 4). This ‘open end’ is not blocked by the bulky charged sidechains (1410–1416) in the linker interacting with the acetylated N-terminus of actin(N−1).

To arrive at a filament-bound structure we superimposed the cryo-electron microscope structures of filamentous actin (PDB code: 6JNO) on both bound actin molecules in Bni1:actin (PDB code: 1Y64) and FMNL3:actin (PDB code: 4EAH) heterodimers. As described in the text, we found in each case that only one of the overlapped molecules in each heterodimer oriented the helical filament axis in a nearly (13° for Bni1) or collinear (FMNL3) direction with the crystal 2-fold screw axis of symmetry relating the bound actin molecules. When the models were aligned, by superposing actin(N−1), actin(N) is in a helically symmetric position with respect to actin(N−1) and actin(N−2). This approach differs fundamentally from previous approaches^24,26^ where initial models for molecular dynamics simulations were constructed by simultaneously docking filament strands on both bound actin molecules and manually establishing linker-lasso interactions to create an open ring state.

Although coplanarity of actin(N) and actin(N−1) is apparent by visual inspection of Figs. 1 and 4, we showed that the average value of the direction cosines of all lines connecting matching backbone atoms of the structural cores^35^ the small domains (small domain residues: 1–38, 68–135, 335–350, 352–375) of Bni1 actin(N) and filamentous actin(N−2) were equal within experimental error (cos α = − 0.0536 ± 0.0382; cos β = 0.9737 ± 0.0098; cos γ = − 0.2133 ± 0.0441), corresponding to angles of α = 93°, β = 13.0°, γ = 102.3°. The variance in the direction cosines increases by 50% if the large domains (subdomains 3 and 4) are included (large domain structural core: residues 150–195, 209–232, 245–320). The treatment of small and large domains separately results in a relative rotation of 5.3° about the shear plane between the small and large structural cores of Bni1 actin(N). This implies that the formin FH2 domain positions the two halves of actin(N) slightly differently at the knob and post ends.

The structure of profilin-β-actin (PDB code: 2BTF) was superposed on the actin molecules bound to the FH2 domains to replace the skeletal isoform with cytoplasmic actin with bound profilin, and to add missing residues at the N- and C- termini and SD2 (residues 32–51). The average RMSD for the polypeptide backbone was 1.24 Å. Profilin can bind with only a small clash (< 0.2 Å) between G117 of bovine profilin and R1423 of FH2(L) at the closed end, which can be relieved by a small backbone rotation at the distant A1401. The FMNL3:actin structure could not accommodate a docked profilin molecule owing to extensive clashes.

The docking of the dimer actin(R)–actin(N+1) to actin(N−1) was carried out by superposing one subunit of the trimer derived from the profilin–β-actin crystal structure (space group 19; PDB code: 2BTF) with actin(N−1). The steps involved in modeling the binding of profilin to the poly-proline tracks in the FH1 sequence are described in the main text. It should be noted, however, that the superpositions, in keeping with the basic strategy of our argument, all involve well-refined, highly conserved, minimum energy structures and interfaces. In particular, the co-crystal structures of mammalian profilin with poly-l-proline^7,54^ allow a series of homologous superpositions from these structures to mammalian profilin in building the transition state model. The pendant profilins on actin(R) and actin(N+1) were positioned on PC and PA in a 90 residue α-helix that required only a very small hinge rotation at a hinge-point at position T1268. The profilins were then superposed with Ena-VASP profilin–PLP co-crystals (PDB codes: 2PAV and 1CJF) to load the profilin(R) and profilin(N+1) poly-l-proline tracts at PC and PA respectively. We overlapped PC with the structure of the pentadecamer bound to the bi-directional dimer (HPP-1 and HPP-2) of human platelet profilin^54^ and chose the molecule with a direction consistent with the position of actin(R) (PDB code:1CJF). The path of the FH2(T) linker is dictated by its interaction with the acetylated N-terminus of actin(N−1) because we did not re-direct it to form a ring from the dimer observed in the co-crystals.

The crystal structures of Bni1–actin and FMNL3–actin represent two states of a common mechanism, yet the angles their knob domains make with actin are not the same^21^. We note that the asymmetric unit of the FMNL3–actin crystal (PDB code: 4EAH) contains two FH2–actin heterodimers (‘biological units’) related by a two-fold non-crystallographic axis^21^. The front-to-back interface between these heterodimers involves a nearly identical set of residues between the knobs of abutting FH2 molecules (V636–A641; A689–L707), burying a total of 1680 Å^2^ (obtained with the PDBePISA suite of interface analysis programs, see Ref.^51^ and https://www.ebi.ac.uk/pdbe/prot_int/pistart.html), and equivalent to a Gibbs Free Energy of 6.1 kcal/mol. This is more than enough to cause the slight separation of the bound actin molecules along the one-start helix and the difference in the angle made between the FMNL3 knob and actin compared with that seen in the FH2-Bni1 structure. The two FMNL3 FH2 knob domains in the asymmetric unit superpose with an RMS = 0.04 Å, and the FH2–actin heterodimers with an all-atom RMS of 0.25 Å.

## Data Availability

The models in this paper are based on structural data deposited in the Rutgers Center for Structural Biology (http://www.rcsb.org).
