# Author Correction: A structural model of the profilin–formin pacemaker system for actin filament elongation

**DOI:** 10.1038/s41598-023-29184-w

**Published:** 2023-02-15

**Authors:** Clarence E. Schutt, Mattias Karlén, Roger Karlsson

**Affiliations:** 1 Department of Chemistry, Princeton University, Princeton, NJ USA; 2 Firma Mattias Karlén, Stockholm, Sweden; 3 Department of Molecular Biosciences, WGI, Stockholm University, Stockholm, Sweden

Correction to: *Scientific Reports* 10.1038/s41598-022-25011-w, published online 28 November 2022

The original version of this Article contained errors in the legends of Figures 1, 2 and 4.

In the legend of Figure 1, the word “trailing” has been corrected to “leading”,

“For consistency in relating the Bni1 and FMNL3 filament-bound structures to a second stepping model, the FH2 domains are labeled so that actin(N) is bound to FH2(L) on the right even though it is not the ‘trailing’ FH2 domain.”

now reads:

“For consistency in relating the Bni1 and FMNL3 filament-bound structures to a second stepping model, the FH2 domains are labeled so that actin(N) is bound to FH2(L) on the right even though it is not the ‘leading’ FH2 domain.”

In the legend of Figure 2, a sentence was missing that described the arrow in Figure 2 A,

Central role for profilin R88 in coordinating actin polymerization by FH2. The boxed region of panel (A) shows profilin residues R88 and H119 sequestering Y169, the keystone residue in the longitudinal actin(N)-actin(N − 2) interface. Actin residues K373, C374 and F375 form part of the pocket surrounding Y169. Panel (B), boxed region, shows (left) the interaction between actin(N) residue D2 and the FH2(L) linker residues K1410 and R1411.

now reads:

Central role for profilin R88 in coordinating actin polymerization by FH2. The boxed region of panel (A) shows profilin residues R88 and H119 sequestering Y169, the keystone residue in the longitudinal actin(N)-actin(N − 2) interface. Actin residues K373, C374 and F375 form part of the pocket surrounding Y169. The arrow points to the prominent profilin ‘wing’ R88-T97 (purple). Panel (B), boxed region, shows (left) the interaction between actin(N) residue D2 and the FH2(L) linker residues K1410 and R1411.

In the legend of Figure 4, actin(R) was incorrectly written as actin(N + 1),

Panel (C) shows selected residues of the clipped structure from the top to reveal the position (red dot) of a three-way interaction between the linker, the N-terminus of actin(N − 1) and the D-loop of actin(N + 1).

now reads:

Panel (C) shows selected residues of the clipped structure from the top to reveal the position (red dot) of a three-way interaction between the linker, the N-terminus of actin(N − 1) and the D-loop of actin(R).

The original Article has been corrected.

